# Emergency medical services utilisation among febrile children attending emergency departments across Europe: an observational multicentre study

**DOI:** 10.1007/s00431-023-05056-3

**Published:** 2023-06-24

**Authors:** Chantal D. Tan, Clementien L. Vermont, Joany M. Zachariasse, Ulrich von Both, Irini Eleftheriou, Marieke Emonts, Michiel van der Flier, Jethro Herberg, Benno Kohlmaier, Michael Levin, Emma Lim, Ian K. Maconochie, Federico Martinon-Torres, Ruud G. Nijman, Marko Pokorn, Irene Rivero-Calle, Maria Tsolia, Werner Zenz, Dace Zavadska, Henriëtte A. Moll, Enitan D. Carrol, On behalf of PERFORM consortium (Personalised Risk assessment in febrile children to optimize Real-life Management across the European Union), Michael Levin, Aubrey Cunnington, Tisham De, Jethro Herberg, Myrsini Kaforou, Victoria Wright, Lucas Baumard, Evangelos Bellos, Giselle D’Souza, Rachel Galassini, Dominic Habgood-Coote, Shea Hamilton, Clive Hoggart, Sara Hourmat, Heather Jackson, Ian Maconochie, Stephanie Menikou, Naomi Lin, Samuel Nichols, Ruud Nijman, Ivonne Pena Paz, Priyen Shah, Ching-Fen Shen, Ortensia Vito, Clare Wilson, Amina Abdulla, Ladan Ali, Sarah Darnell, Rikke Jorgensen, Sobia Mustafa, Salina Persand, Molly Stevens, Eunjung Kim, Benjamin Pierce, Katy Fidler, Julia Dudley, Vivien Richmond, Emma Tavliavini, Ching-Chuan Liu, Shih-Min Wang, Federico Martinón-Torres, Antonio Salas, Fernando Álvez González, Cristina Balo Farto, Ruth Barral-Arca, María Barreiro Castro, Xabier Bello, Mirian Ben García, Sandra Carnota, Miriam Cebey-López, María José CurrasTuala, Carlos Durán Suárez, Luisa García Vicente, Alberto Gómez-Carballa, Jose Gómez Rial, Pilar Leboráns Iglesias, Federico Martinón-Torres, Nazareth Martinón-Torres, José María Martinón Sánchez, Belén Mosquera Pérez, Jacobo Pardo-Seco, Lidia Piñeiro Rodríguez, Sara Pischedda, Sara Rey Vázquez, Irene Rivero Calle, Carmen Rodríguez-Tenreiro, Lorenzo Redondo-Collazo, Miguel Sadiki Ora, Sonia Serén Fernández, Cristina Serén Trasorras, Marisol Vilas Iglesias, Dace Zavadska, Anda Balode, Arta Bārzdiņa, Dārta Deksne, Dace Gardovska, Dagne Grāvele, Ilze Grope, Anija Meiere, Ieva Nokalna, Jana Pavāre, Zanda Pučuka, Katrīna Selecka, Aleksandra Sidorova, Dace Svile, Urzula Nora Urbāne, Effua Usuf, Kalifa Bojang, Syed M. A. Zaman, Fatou Secka, Suzanne Anderson, Anna RocaIsatou Sarr, Momodou Saidykhan, Saffiatou Darboe, Samba Ceesay, Umberto D’alessandro, Henriëtte A. Moll, Dorine M. Borensztajn¹, Nienke N. Hagedoorn, Chantal Tan, Clementien L. Vermont, Joany Zachariasse, W Dik, Philipp Agyeman, Luregn J Schlapbach, Christoph Aebi, Verena Wyss, Mariama Usman, Eric Giannoni, Martin Stocker, Klara M Posfay-Barbe, Ulrich Heininger, Sara Bernhard-Stirnemann, Anita Niederer-Loher, Christian Kahlert, Giancarlo Natalucci, Christa Relly, Thomas Riedel, Christoph Aebi, Christoph Berger, Enitan D Carrol, Stéphane Paulus, Elizabeth Cocklin, Rebecca Jennings, Joanne Johnston, Simon Leigh, Karen Newall, Sam Romaine, Maria Tsolia, Irini Eleftheriou, Maria Tambouratzi, Antonis Marmarinos, Marietta Xagorari, Kelly Syggelou, Colin Fink, Marie Voice, Leo Calvo-Bado, Werner Zenz, Benno Kohlmaier, Nina A. Schweintzger, Manfred G. Sagmeister, Daniela S. Kohlfürst, Christoph Zurl, Alexander Binder, Susanne Hösele, Manuel Leitner, Lena Pölz, Glorija Rajic, Sebastian Bauchinger, Hinrich Baumgart, Martin Benesch, Astrid Ceolotto, Ernst Eber, Siegfried Gallistl, Gunther Gores, Harald Haidl, Almuthe Hauer, Christa Hude, Markus Keldorfer, Larissa Krenn, Heidemarie Pilch, Andreas Pfleger, Klaus Pfurtscheller, Gudrun Nordberg, Tobias Niedrist, Siegfried Rödl, Andrea Skrabl-Baumgartner, Matthias Sperl, Laura Stampfer, Volker Strenger, Holger Till, Andreas Trobisch, Sabine Löffler, Shunmay Yeung, Juan Emmanuel Dewez, Martin Hibberd, David Bath, Alec Miners, Ruud Nijman, Catherine Wedderburn, Anne Meierford, Baptiste Leurent, Ronald de Groot, Michiel van der Flier, Marien I. de Jonge, Koen van Aerde, Wynand Alkema, Bryan van den Broek, Jolein Gloerich, Alain J. van Gool, Stefanie Henriet, Martijn Huijnen, Ria Philipsen, Esther Willems, G.P.J.M. Gerrits, M. van Leur, J. Heidema, L. de Haan, C.J. Miedema, C. Neeleman, C.C. Obihara, G.A. Tramper-Stranders, Andrew J. Pollard, Rama Kandasamy, Stéphane Paulus, Michael J. Carter, Daniel O’Connor, Sagida Bibi, Dominic F. Kelly, Meeru Gurung, Stephen Thorson, Imran Ansari, David R. Murdoch, Shrijana Shrestha, Zoe Oliver, Marieke Emonts, Emma Lim, Lucille Valentine, Karen Allen, Kathryn Bell, Adora Chan, Stephen Crulley, Kirsty Devine, Daniel Fabian, Sharon King, Paul McAlinden, Sam McDonald, Anne McDonnell, Ailsa Pickering, Evelyn Thomson, Amanda Wood, Diane Wallia, Phil Woodsford, Frances Baxter, Ashley Bell, Mathew Rhodes, Rachel Agbeko, Christine Mackerness, Bryan Baas, Lieke Kloosterhuis, Wilma Oosthoek, Tasnim Arif, Joshua Bennet, Kalvin Collings, Ilona van der Giessen, Alex Martin, Aqeela Rashid, Emily Rowlands, Gabriella de Vries, Fabian van der Velden, Lucille Valentine, Mike Martin, Ravi Mistry, Ulrich von Both, Laura Kolberg, Manuela Zwerenz, Judith Buschbeck, Christoph Bidlingmaier, Vera Binder, Katharina Danhauser, Nikolaus Haas, Matthias Griese, Tobias Feuchtinger, Julia Keil, Matthias Kappler, Eberhard Lurz, Georg Muench, Karl Reiter, Carola Schoen, François Mallet, Karen Brengel-Pesce, Alexandre Pachot, Marine Mommert, Marko Pokorn, Mojca Kolnik, Katarina Vincek, Tina Plankar Srovin, Natalija Bahovec, Petra Prunk, Veronika Osterman, Tanja Avramoska, Taco Kuijpers, Ilse Jongerius, J.M. van den Berg, D. Schonenberg, A.M. Barendregt, D. Pajkrt, M. van der Kuip, A.M. van Furth, Evelien Sprenkeler, Judith Zandstra, G. van Mierlo, J. Geissler

**Affiliations:** 1 Department of General Paediatrics, Erasmus MC-Sophia Children’s Hospital, Rotterdam, the Netherlands; 2 Section of Paediatric Infectious Diseases, Imperial College, London, UK; 3 Department of Paediatrics, Children Clinical University Hospital, Rīgas Stradiņa Universitāte, Riga, Latvia; 4 Division of Paediatric Infectious Diseases, Dr. Von Hauner Children’s Hospital, University Hospital, Ludwig-Maximilians University, Munich, Germany; 5 German Centre for Infection Research, DZIF, Partner Site, Munich, Germany; 6 Institute of Infection, Veterinary and Ecological Sciences, University of Liverpool, Liverpool, UK; 7 Alder Hey Children’s NHS Foundation Trust, Liverpool, UK; 8 Second Department of Paediatrics, P. and A. Kyriakou Children’s Hospital, National and Kapodistrian University of Athens, Athens, Greece; 9 Paediatric Immunology, Infectious Diseases & Allergy, Great North Children’s Hospital, Newcastle Upon Tyne Hospitals NHS Foundation Trust, Newcastle Upon Tyne, UK; 10 Translational and Clinical Research Institute, Newcastle University, Newcastle Upon Tyne, UK; 11 Section of Paediatric Infectious Diseases, Laboratory of Medical Immunology, Radboud Center for Infectious Diseases, Radboud Institute for Molecular Life Sciences, RadboudUMC, Nijmegen, the Netherlands; 12 Paediatric Infectious Diseases and Immunology, Amalia Children’s Hospital, RadboudUMC, Nijmegen, the Netherlands; 13 Paediatric Infectious Diseases and Immunology, Wilhelmina Children’s Hospital, University Medical Centre Utrecht, Utrecht, The Netherlands; 14 Department of General Paediatrics, Medical University of Graz, Graz, Austria; 15 Paediatric Emergency Medicine, Imperial College Healthcare Trust NHS, London, UK; 16 Genetics, Vaccines, Infections and Paediatrics Research Group (GENVIP), Hospital Clínico Universitario de Santiago de Compostela, Santiago de Compostela, Spain; 17 Department of Infectious Diseases and Faculty of Medicine, University Medical Centre Ljubljana, University of Ljubljana, Ljubljana, Slovenia; 18 Department of Paediatric Infectious Diseases and Immunology, Erasmus MC-Sophia Children’s Hospital, Rotterdam, the Netherlands; 19 NIHR Newcastle Biomedical Research Centre, Newcastle Upon Tyne Hospitals NHS Trust, Westgate Rd, Newcastle Upon Tyne, NE4 5PL UK; 20 Department of Medicine, Population Health Sciences Institute, Newcastle University, Newcastle Upon Tyne, UK

**Keywords:** Emergency medical services, Emergency care, Children, Fever, Paediatrics

## Abstract

Children constitute 6–10% of all patients attending the emergency department (ED) by emergency medical services (EMS). However, discordant EMS use in children occurs in 37–61% with fever as an important risk factor. We aimed to describe EMS utilisation among febrile children attending European EDs. This study is part of an observational multicentre study assessing management and outcome in febrile children up to 18 years (MOFICHE) attending twelve EDs in eight European countries. Discordant EMS use was defined as the absence of markers of urgency including intermediate/high triage urgency, advanced diagnostics, treatment, and admission in children transferred by EMS. Multivariable logistic regression analyses were performed for the association between (1) EMS use and markers of urgency, and (2) patient characteristics and discordant EMS use after adjusting all analyses for the covariates age, gender, visiting hours, presenting symptoms, and ED setting. A total of 5464 (15%, range 0.1–42%) children attended the ED by EMS. Markers of urgency were more frequently present in the EMS group compared with the non-EMS group. Discordant EMS use occurred in 1601 children (29%, range 1–59%). Age and gender were not associated with discordant EMS use, whereas neurological symptoms were associated with less discordant EMS use (aOR 0.2, 95%CI 0.1–0.2), and attendance out of office hours was associated with more discordant EMS use (aOR 1.6, 95%CI 1.4–1.9). Settings with higher percentage of self-referrals to the ED had more discordant EMS use (*p* < 0.05).

*  Conclusion*: There is large practice variation in EMS use in febrile children attending European EDs. Markers of urgency were more frequently present in children in the EMS group. However, discordant EMS use occurred in 29%. Further research is needed on non-medical factors influencing discordant EMS use in febrile children across Europe, so that pre-emptive strategies can be implemented.

**Table:** 

**What is Known:**
•*Children constitute around 6–10% of all patients attending the emergency department by emergency medical services.*
•*Discordant EMS use occurs in 37–61% of all children, with fever as most common presenting symptom for discordant EMS use in children.*
**What is New:**
•*There is large practice variation in EMS use among febrile children across Europe with discordance EMS use occurring in 29% (range 1–59%), which was associated with attendance during out of office hours and with settings with higher percentage of self-referrals to the ED.*
•*Future research is needed focusing on non-medical factors (socioeconomic status, parental preferences and past experience, healthcare systems, referral pathways, out of hours services provision) that influence discordant EMS use in febrile children across Europe.*

**Supplementary Information:**

The online version contains supplementary material available at 10.1007/s00431-023-05056-3.

## Introduction

Emergency medical services (EMS) provide urgent out-of-hospital treatment and stabilisation for serious illness and injury, and transport patients to the emergency department (ED) for definitive care [1]. It was primarily developed to provide immediate care to adults with traumatic injury or cardiac arrest for whom fast intervention or transport is lifesaving [2, 3]. However, in the 1980s children were acknowledged as a representative group of paramedic calls and constituted 6–10% of all patients transported to the ED by EMS [2–5]. The majority of children are in the trauma category, but unlike adults, fever and reassurance are common reasons for EMS use [5, 6]. Fever in children accounts for 20% of all paediatric emergency visits with the majority having a self-limiting viral respiratory illness [7, 8].

Currently, there is a high pressure on EMS and EDs are overcrowded [9, 10]. Therefore, it is important that EMS is only used by patients who truly need this [11]. In literature there is no universal definition for discordant EMS use and most often discordance is assigned retrospectively by clinicians or based on resource utilisation [6, 12]. The estimated prevalence of discordant EMS use in children is 37–61% [13, 14]. Risk factors for discordant EMS use in children are lack of private transport, being first-time parents, low level of parental education, and parental anxiety [6]. Fever is reported as the most common presenting symptom for discordant EMS use in children [15]. This might imply that it is difficult for parents and healthcare professionals to assess disease severity in febrile children. Furthermore, discordant EMS use can also be influenced by non-medical factors such as the healthcare system in place [16].

Discordant EMS use leads to high medical costs, shortage of EMS, and high work pressure for EMS and ED staff [17]. Data from a large international cohort on febrile children attending EDs by EMS is lacking, and discordant EMS use might occur [15]. Therefore, the aim of this study is to describe EMS utilisation in febrile children attending EDs across Europe.

## Methods

### Study design and setting

This is a secondary analysis of the MOFICHE study (Management and Outcome of Fever in children in Europe), which is part of the PERFORM project (Personalized Risk assessment in Febrile illness to Optimize Real-life Management across the European Union) [18, 19]. The MOFICHE study is an observational multicentre study evaluating management and outcome of febrile children in twelve EDs across eight European countries (Austria, Germany, Greece, Latvia, the Netherlands *n* = 3, Slovenia, Spain, UK *n* = 3). The hospital characteristics are described in a previous study [20] (Appendix A). Approval by the ethics committees of the participating hospitals was obtained. The need for informed consent was waived and in the UK settings an additional opt-out mechanism was in place.

### Study population

Children < 18 years with fever (≥ 38 ℃) at the ED or with a history of fever (within 3 days before ED visit) were included in MOFICHE. For this secondary analysis, febrile children with a known way of transportation defined as EMS versus non-EMS were included. Children with missing data on triage urgency and admission were excluded.

### Data collection

Data of 38,480 febrile children attending the ED were collected as part of routine clinical care and extracted from electronic health records by the local research team (January 2017–April 2018). Data collected included patient characteristics (age, gender, presenting symptoms, comorbidity (chronic condition expected to last at least 1 year [21])), referral status, visiting hours, markers of disease severity (triage urgency, vital signs, ill appearance), diagnostics (laboratory tests, imaging), treatment (immediate life-saving interventions (ILSI), oxygen therapy), and disposition (admission to ward or paediatric intensive care unit (PICU)). Focus of infection and cause of infection were retrospectively assigned by the research team. Focus of infection was categorised into respiratory tract, gastrointestinal tract, urinary tract, sepsis/meningitis, and other (e.g. skin/soft tissue infection, undifferentiated fever, inflammatory illness). Cause of infection was categorised into presumed bacterial, unknown bacterial/viral, presumed, viral and other according to a previously published phenotyping flowchart [19] (Appendix B). Presenting symptoms were categorised into neurological, respiratory, gastrointestinal, and other symptoms. Neurological symptoms included meningeal signs, focal neurological signs, or seizures. Respiratory symptoms included coughing or other signs of respiratory tract infection, such as runny nose, sore throat, and sneezing. Gastrointestinal symptoms included vomiting or diarrhoea. Children with more than one presenting symptom were classified according to a priority system with neurological symptoms having priority. If children had both respiratory and gastrointestinal symptoms, they were categorised based on focus of infection, meaning that respiratory tract infections were allocated to the respiratory group and children with a gastrointestinal focus of infection were allocated to the gastrointestinal group. If children had another focus of infection, none of these three symptom groups, or missing data on these three symptom groups, they were categorised as other. Out of office hours (OOH) was defined as between 5 PM and 8AM on weekdays or the weekends. Referral status was categorised into EMS, referral by a medical specialist or general practitioner (GP), other (e.g. referral by other hospital), and self-referral. Triage urgency was divided into low and intermediate/high triage urgencies. Abnormal vital signs, including tachypnoea, tachycardia, and hypoxia, were based on age-dependent cut-off values according to the Advanced Pediatric Life Support guideline of 2017 [22]. Diagnostic tests were categorised into simple and advanced diagnostics. Simple diagnostics included C-reactive protein (CRP), white blood cell (WBC) count, urinalysis, urine culture, and respiratory test or culture. Advanced diagnostics included any kind of imaging, blood culture, and lumbar puncture. ILSI consist of five categories [23] (Appendix C).

For this substudy, a questionnaire on background information on EMS was sent out to the principal investigator per country. This questionnaire contributed to additional qualitative information next to the clinical information in MOFICHE. It consisted of one open question and four multiple choice questions concerning how the whole EMS process looks like, who can call the emergency number, what happens when EMS arrive at the patient, where the EMS can transport the patient to, and what the practical reasons for EMS use are (Appendix D).

### Outcome measures

We defined way of transportation as transport by EMS versus non-EMS, which we created based on registered referral status. The non-EMS group contained the other categories of referral status. The definition of discordant EMS use was based on previous literature and expert opinions of the local research team including paediatricians [6]. Discordant EMS use was defined as the absence of markers of urgency including intermediate/high triage urgency, advanced diagnostics, oxygen therapy, ILSI, and admission. If none of these five criteria was met, EMS use was defined as discordant.

### Data analysis

Descriptive statistics were used for patient characteristics and management stratified by EMS use. We performed Mann–Whitney U and chi-squared testing for continuous and categorical data assuming not normally distributed data. Multivariable logistic regression analysis was performed for the association between EMS use and markers of urgency with adjustment for the confounders age, gender, visiting hours, presenting symptoms, and ED setting. Additionally, multivariable logistic regression analysis was performed for the association between patient characteristics (age, gender, presenting symptoms, and visiting hours) and discordant EMS use. We created four age categories: < 1, 1 < 5, 5 < 12, 12 < 18 years. Results are shown as adjusted odds ratios (aOR) with a 95% confidence interval (CI). We explored the association between discordant EMS use and (1) markers of urgency and (2) proportion self-referrals as we hypothesised these as important predictors of discordant EMS use. A heat map was created on discordant EMS use and markers of urgency per ED setting, and chi-squared testing was used for the hospital characteristic self-referral rate and discordant EMS use. ED settings were dichotomised in high versus low self-referral ED settings based on the prevalence of 57% self-referrals in the MOFICHE population. A sensitivity analysis was performed to simulate the situation where 20% of EMS transports are initiated by other healthcare professionals, e.g. the GP [24]. The percentage of discordant EMS use was then calculated in a random 80% children per ED setting. Statistical analyses were performed using SPSS software version 25 with a *p*-value below 0.05 considered statistically significant. Qualitative data are reported descriptively to interpret the findings of the quantitative analyses.

## Results

### Patient characteristics

A total of 37,315 children were included, and 36,156 children were used for analyses after exclusion of children with missing data on triage urgency (3%) and hospital admission (0.1%). Fifteen percent (*N* = 5464) of the children were transported by EMS (range 0.1–42%) (Appendix F). Children in the EMS group more often attended the ED during OOH (76% vs 65%), were more frequently triaged as intermediate/high urgent (56% vs 32%), described as ill appearing (29% vs 13%) and had more abnormal vital signs compared to children in the non-EMS group. Neurological symptoms were more common in the EMS group, whereas respiratory symptoms were more common in the non-EMS group. The EMS group received more extensive management in terms of diagnostic tests, treatment, and admission. Focus and cause of infection were comparable in the two groups (Table 1).

**Table 1:** Patient characteristics and management stratified by EMS use (*N* = 36,156)

	**EMS**	**Non-EMS **	**Missing**
	***N*** **= 5464 (15)**	***N*** **= 30,692 (85)**	
Age (years)^a^*	2.4 (1.3–4.9)	2.8 (1.3–5.7)	/
Gender (male)	3012 (55)	16,845 (55)	1 (0)
Comorbidity*	745 (14)	5167 (17)	343 (0.9)
Visiting hours (out of office)*	4152 (76)	19,912 (65)	/
Triage urgency*			/
* Low*	2427 (44)	20,890 (68)	
* Intermediate/high*	3037 (56)	9802 (32)	
Ill appearance*	1591 (29)	4050 (13)	1507 (4)
Abnormal vital signs			
Tachycardia*	1594 (29)	7568 (25)	3134 (9)
Tachypnoea*	1095 (20)	4407 (14)	7664 (21)
* Hypoxia*	113 (2)	683 (2)	4436 (12)
Presenting symptoms*			/
* Neurological*	740 (14)	772 (3)	
*Respiratory*	2580 (47)	17,975 (59)	
* Gastrointestinal*	829 (15)	4727 (15)	
* Other*	1315 (24)	7218 (24)	
Diagnostics*	4000 (73)	18,259 (60)	/
Simple*	3861 (71)	17,252 (56)	
* CRP*	3301 (60)	12,444 (41)	
* WBC count*	3290 (60)	12,370 (40)	
* Urine analysis*	1851 (34)	7113 (23)	
* Urine culture*	268 (5)	2446 (8)	
* Respiratory test*	919 (17)	5329 (17)	
Advanced*	1431 (26)	7012 (23)	
* Imaging*	1279 (23)	5326 (17)	
* Chest X-ray*	1008 (18)	4158 (14)	
* Other X-ray*	111 (2)	327 (1)	
* Ultrasound*	229 (4)	1170 (4)	
* CT*	54 (1)	141 (0.5)	
* MRI*	10 (0.2)	71 (0.2)	
* Blood culture*	340 (6)	3016 (10)	
* Lumbar puncture*	53 (1)	351 (1)	
Treatment			/
Oxygen therapy*	209 (4)	752 (3)	
ILSI^*^	204 (4)	386 (1)	
Hospital admission*	1955 (36)	7310 (24)	
* To ward*	1911 (35)	7212 (24)	
* To PICU*	44 (0.8)	98 (0.3)	
Focus of infection*			5 (0)
* Sepsis/meningitis*	31 (0.6)	231 (0.8)	
* Respiratory tract*	3816 (70)	20,492 (67)	
* Gastrointestinal tract*	629 (12)	3163 (10)	
* Urinary tract*	197 (4)	1042 (3)	
* Other*	775 (14)	5760 (19)	
Cause of infection*			247 (0.7)
* Presumed bacterial*	1360 (25)	6580 (21)	
* Unknown bacterial/viral*	699 (13)	4764 (16)	
* Presumed viral*	3159 (58)	17,143 (56)	
* Other*	234 (4)	1970 (6)	

Absolute numbers and percentages (%) are shown

*ILSI* immediate life-saving interventions on arrival at the ED, *PICU* paediatric intensive care unit

*Significantly differed between the EMS and non-EMS group (*p*-value < 0.05)

^a^Median and interquartile range 25–75 are shown

**Fig. 1:**
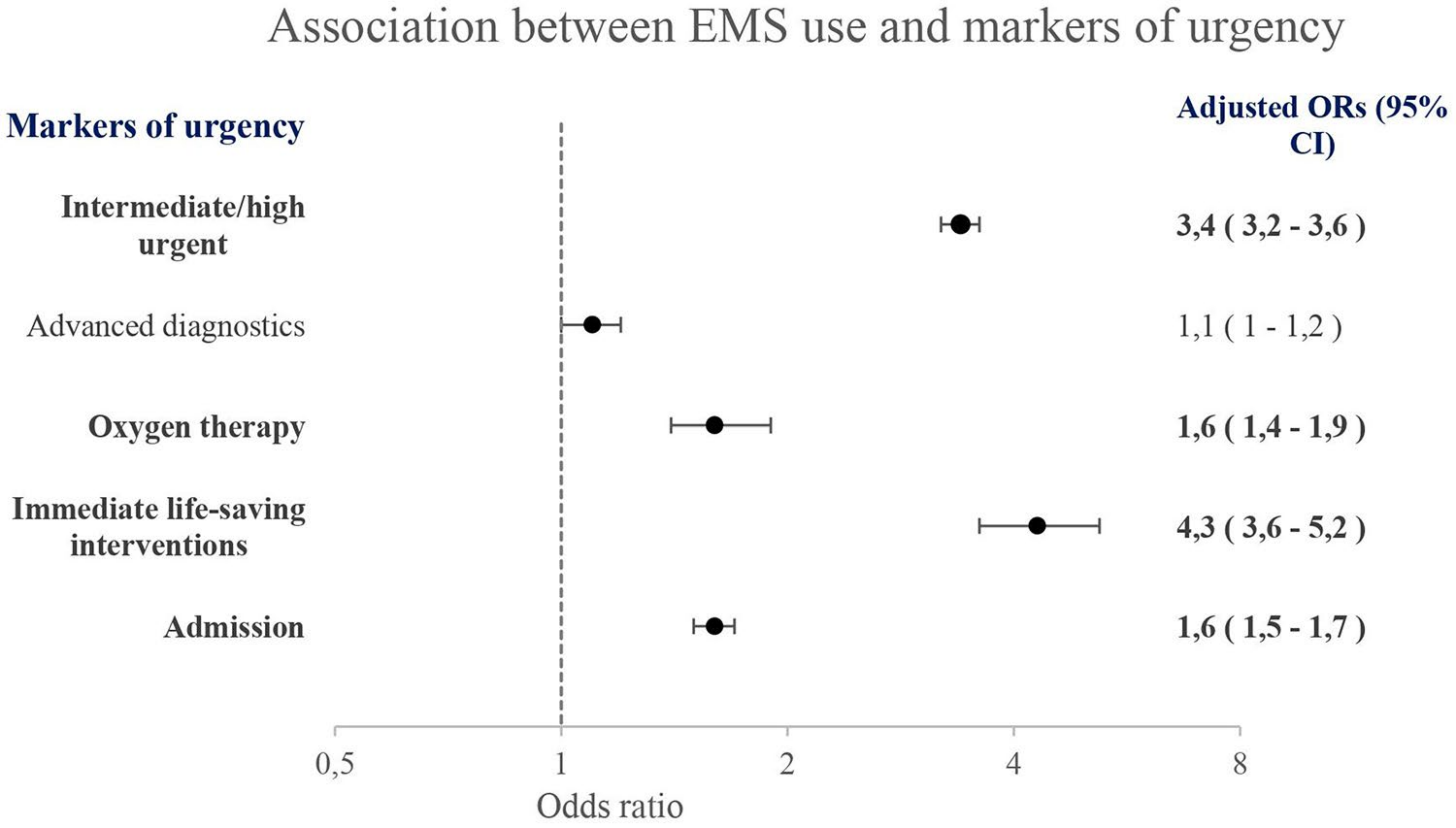
Association between EMS use and markers of urgency as five separate models with the non-EMS group as reference group. Adjusted for age, gender, visiting hours, presenting symptoms, and ED setting

Four markers of urgency including intermediate/high triage urgency, oxygen therapy, ILSI, and admission were significantly more frequently present in the EMS group compared to the non-EMS group. Advanced diagnostics was comparable in the EMS group and the non-EMS group (Fig. 1, Appendix G).

### Discordant EMS use

**Fig. 2:**
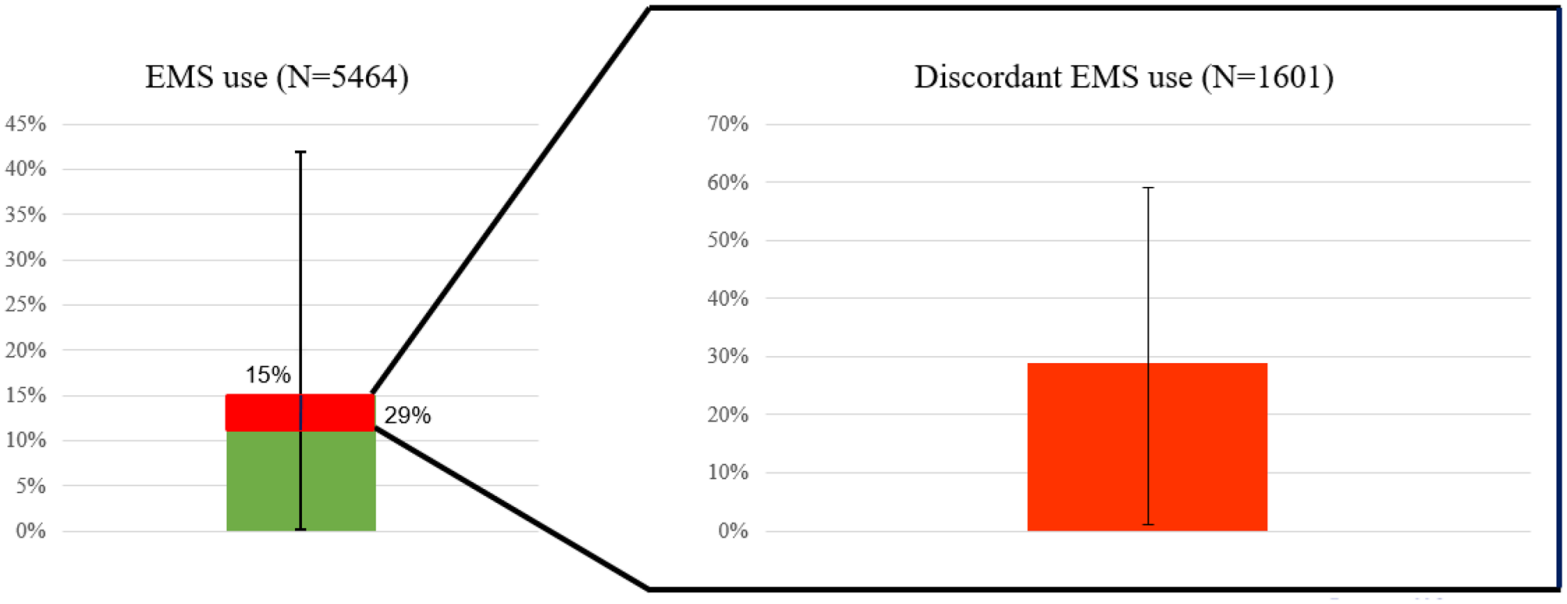
Proportion EMS use (15%, *N* = 5464/36,256) and discordant EMS use (29%, *N* = 1601/5464) with corresponding ranges across the EDs depicted with error bars

Of the 5464 children who were transported by EMS, 1601 children (29%) had discordant EMS use ranging from 1 to 59% across the EDs (Fig. 2, Appendix F). Since the most important markers of urgency are intermediate/high triage urgency and admission, we first evaluated the proportion of discordant EMS use by defining discordant EMS use as the absence of these two main markers of urgency separately. This resulted in 2427 children (44%) and 3509 children (64%), respectively, having discordant EMS use. When combining the absence of these two markers of urgency for the definition of discordant EMS use, 1865 children (34%) had discordant EMS use. Lastly, when the other three markers of urgency (advanced diagnostics, oxygen therapy, ILSI) were added, the proportion discordant EMS use was 29% (range 1–59%).

In EDs with more markers of urgency present, the proportion of discordant EMS use was low, whereas EDs with less markers of urgency present having more discordant EMS (Table 2). ED settings with high self-referral rates to the ED (> 57%) were having more discordant EMS use (*p* < 0.05) (Appendix A). A sensitivity analysis using a random 80% children per ED setting did not show any differences in discordant EMS use (Appendix H).

**Table 2:**
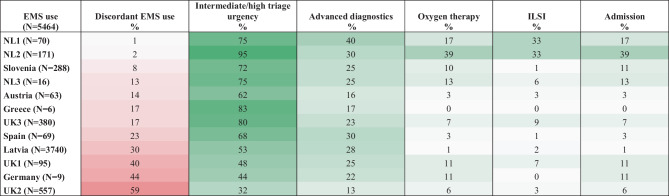
Heat map of discordant EMS use and markers of urgency per ED

Percentages per ED are shown. Red shades depict discordant EMS use. Green shades depict markers of urgency

Children in the younger age groups tended to have more discordant EMS use compared with children 12 < 18 years old. Gender was not associated with EMS use (aOR 1.1, 95% CI 1.0–1.3). The neurological group had less discordant EMS use with an aOR of 0.2 (95% CI 0.1–0.2). ED attendance during OOH was associated with more discordant EMS use (aOR 1.6, 95% CI 1.4–1.9) (Table 3).

**Table 3:** Association between patient characteristics and discordant EMS use

	**OR (95% CI)**	**aOR (95% CI)**
Age (years)^a^		
*< 1*	1.4 (1.1–1.8)	1.4 (1.1–1.8)
*1 < 5*	1.3 (1.0–1.7)	1.3 (1.0–1.7)
*5 < 12*	1.4 (1.1–1.9)	1.5 (1.1–1.9)
*12 < 18*	REF	REF
Gender		
*Girls*	1.1 (1.0–1.2)	1.1 (1.0–1.3)
*Boys*	REF	REF
Presenting symptoms		
*Neurological*	0.2 (0.1–0.3)	0.2 (0.1–0.2)
*Respiratory*	0.9 (0.8–1.1)	0.9 (0.7–1.0)
*Gastrointestinal*	0.8 (0.6–0.9)	0.8 (0.6–0.9)
*Other*	REF	REF
Visiting hours		
*OOH*	1.6 (1.4–1.9)	1.6 (1.4-–0.9)
*During office hours*	REF	REF

*OOH* out of office hours

^a^Age distribution per setting is shown in Appendix I

### EMS process per country

The EMS process in all participating countries is rather comparable: calling the emergency number by anyone is possible, triage and assessment by phone to decide whether EMS transport is needed, assessment by EMS staff and decision on transport to the ED or stay at home, or advice GP consultation. In Spain, patients can be transported to the GP OOH by EMS as well, and in Greece any child will be transferred to the ED if parents are patient enough to wait. The reasons for EMS use are not only transport for critically ill patients but also lack of private transport and an emergency call during OOH. The full answers on the questionnaire are shown in Appendix D and a diagram of healthcare pathways initiating EMS transport to the ED is shown in Appendix E.

## Discussion

### Main findings

There is large practice variation in EMS use in febrile children across the participating EDs, ranging from 0.1 to 42%. Children arriving by EMS are more often triaged as high urgent and received more extensive management. On one hand, this could be explained by the fact the children arriving by EMS are more severely ill and therefore receive more diagnostics tests and get admitted more often. On the other hand, physicians may be steered towards a more aggressive workup by the transportation by EMS itself. A previous study on diagnostic variation in febrile children attending European EDs showed that having a high triage urgency level leads to more diagnostic testing and hospitalisation after adjusting for patient and hospital characteristics [25]. In our febrile paediatric population, discordant EMS use occurred in 29% ranging from 1 to 59% assuming differences in patient mix, prehospital pathways, and healthcare systems. We found no clear association between age and gender and discordant EMS use. However, children presenting with neurological symptoms were associated with less discordant EMS use (aOR 0.2, 95% CI 0.1–0.2), which was also reported in a previous study with seizures or altered mental status accounting for the lowest percentage of discordant EMS use, while fever accounted for the highest percentage [15]. By contrast, EMS transport during OOH hours was associated with more discordant EMS use (aOR 1.6, 95% CI 1.4–1.9), possibly reflecting differences in prehospital healthcare systems (e.g. availability of GP OOH) and social factors as socioeconomic status [20]. Similarly, settings with high self-referral rates to the ED had more discordant EMS use, assuming referral pathways as important predictor for EMS use. Lastly, our questionnaire showed that the EMS process in the participating countries is quite similar, and therefore we could not use this information for the analysis. This implies that there are other factors accounting for the large range in (discordant) EMS use. However, we did not have information on non-medical factors, e.g. access to healthcare, socioeconomic status, distance from hospital, availability of GP OOH, language barriers, and public transport [14].

### Strengths and limitations

This is the first study examining the use of EMS and the proportion discordant EMS use in febrile children across eight European countries, and it discusses an important current medical topic. [9, 10] The MOFICHE study involved extensive data collection on patient characteristics, disease severity, and management. A clear definition of discordant EMS use was created based on extensive literature search and input of the research team. The need for a universal definition for dis/concordant EMS use was already mentioned in a recent systematic review from Proctor et al. on factors associated with ambulance use for non-emergency problems in children. They stated that “Both policymakers and academics need to work towards a contextually nuanced and consistent definition of ‘appropriate’ ambulance resource use” [6]. There are several limitations that should be acknowledged as well. Since the MOFICHE study includes data from large European university hospitals, the results may not be generalisable to smaller general hospitals. We have not taken into account non-medical factors that could influence the choice for transport by EMS and the proportion discordant EMS use, such as long arrival time due to geographical location when living in a rural area or socioeconomic status [26, 27]. Lastly, there might have been misclassification of the EMS group as we had no information on who initiated the EMS pathway. However, a sensitivity analysis in which we simulated that 20% of EMS transports was initiated by GPs showed no change in the proportion discordant EMS use.

### Implications for clinical practice and future research

Practice variation in both EMS use and discordant EMS use across Europe can be attributable to non-medical factors, such as healthcare system–related (e.g. referral systems, availability of resources, OOH emergency provision) and societal factors (e.g. availability of transport, socioeconomic status, parental experiences, distance to hospital). Qualitative research is needed to explore the reasons of parents to call EMS for their febrile child [28]. An eHealth tool is being developed for parents to support them assessing their febrile child’s disease severity based on alarm symptoms such as lethargy [29]. Further research is needed on non-medical factors influencing discordant EMS use and refining healthcare pathways might lead to more efficient EMS use. It would be valuable to examine the influence of longer GP’s office hours and the availability of OOH GPs with specific experience in paediatrics.

## Conclusion

There is large practice variation in EMS use in febrile children attending European EDs. Markers of urgency were more frequently present in children attending the ED by EMS. However, discordant EMS use occurred in 29%, ranging from 1 to 59% across EDs. Age and gender were not associated with discordant EMS use, whereas neurological symptoms were associated with less discordant EMS use and attendance during OOH was associated with more discordant EMS use. Settings with higher self-referral rates to the ED had more discordant EMS use. Further research is needed to understand non-medical factors influencing discordant EMS use in febrile children across Europe, so that pre-emptive strategies can be implemented.

### Supplementary Information

Below is the link to the electronic supplementary material.

Supplementary file1 (PDF 756 KB)

## Data Availability

Data of this study are available upon request from the corresponding author (edcarrol@liverpool.ac.uk), subject to local rules and regulations.

## Abbreviations

aOR: Adjusted odds ratio
CI: Confidence interval
CRP: C-reactive protein
ED: Emergency department
GP: General practitioner
ILSI: Immediate life-saving interventions
MOFICHE: Management and Outcome of Fever in children in Europe
OOH: Out of office hours
PERFORM: Personalized Risk assessment in Febrile illness to Optimize Real-life Management across the European Union
PICU: Paediatric intensive care unit
WBC: White blood cell
UK: United Kingdom
