# Supplementary material for: Emergency medical services utilisation among febrile children attending emergency departments across Europe: an observational multicentre study
Source: Eur J Pediatr. 2023 Jun 24;182(9):3939–47. doi: 10.1007/s00431-023-05056-3 (PMC10570223; doi:10.1007/s00431-023-05056-3)
Supplement: Supplementary file 1 — Supplementary file1 (PDF 756 KB) [file 431_2023_5056_MOESM1_ESM.pdf]

## Supplementary files

**Article title:** Emergency Medical Services utilisation among febrile children attending Emergency Departments across Europe: an observational multicenter study

**Journal Name:** European Journal of Pediatrics


**Author names:**
Chantal D. Tan[1], Clementien L. Vermont[17], Joany M. Zachariasse[1], Ulrich von Both[4,5], Irini Eleftheriou[8], Marieke Emonts[9,10,18], Michiel van der Flier[11,11A,12], Jethro Herberg[2], Benno Kohlmaier[13], Michael Levin[2], Emma Lim[9,19], Ian K. Maconochie[14], Federico Martinon-Torres[15], Ruud G. Nijman[2], Marko Pokorn[16], Irene Rivero-Calle[15], Maria Tsolia[8], Werner Zenz[13], Dace Zavadska[3], Henriëtte A. Moll[1], Enitan D. Carrol[6,7]

**Author affiliations:**
1. Erasmus MC-Sophia Children's Hospital, Department of General Paediatrics, Rotterdam, the Netherlands
2. Section of Paediatric Infectious Diseases, Imperial College, London, United Kingdom
3. Rīgas Stradiņa universitāte, Department of Paediatrics, Children clinical university hospital, Riga, Latvia
4. Division of Paediatric Infectious Diseases, Dr. von Hauner Children's Hospital, University Hospital, Ludwig-Maximilians-University Munich, Germany
5. German Centre for Infection Research, DZIF, partner site Munich, Germany
6. University of Liverpool, Institute of Infection, Veterinary and Ecological Sciences Liverpool, United Kingdom
7. Alder Hey Children's NHS Foundation Trust, Liverpool, United Kingdom
8. National and Kapodistrian University of Athens, Second Department of Paediatrics, P. and A. Kyriakou Children's Hospital, Athens, Greece
9. Great North Children's Hospital, Paediatric Immunology, Infectious Diseases & Allergy, Newcastle upon Tyne Hospitals NHS Foundation Trust, Newcastle upon Tyne, United Kingdom
10. Translational and Clinical Research Institute, Newcastle University, Newcastle upon Tyne, United Kingdom
11. Section of Paediatric Infectious Diseases, Laboratory of Medical Immunology, Radboud Center for Infectious Diseases, Radboud Institute for Molecular Life Sciences, RadboudUMC, Nijmegen, the Netherlands
11A Paediatric Infectious Diseases and Immunology, Amalia Children's Hospital, RadboudUMC, Nijmegen, the Netherlands
12. Paediatric Infectious Diseases and Immunology, Wilhelmina Children's Hospital, University Medical Centre Utrecht, Utrecht, The Netherlands
13. Medical University of Graz, Department of General Paediatrics, Graz, Austria
14. Paediatric Emergency Medicine, Imperial College Healthcare Trust NHS, London, United Kingdom
15. Hospital Clínico Universitario de Santiago de Compostela. Genetics, Vaccines, Infections and Paediatrics Research group (GENVIP), Santiago de Compostela, Spain
16. University Medical Centre Ljubljana, Department of Infectious Diseases and Faculty of Medicine, University of Ljubljana, Ljubljana, Slovenia

17. Erasmus MC-Sophia Children's Hospital, Department of Paediatric Infectious diseases and Immunology, Rotterdam, the Netherlands
18. NIHR Newcastle Biomedical Research Centre based at Newcastle upon Tyne Hospitals NHS Trust and Newcastle University, Westgate Rd, Newcastle upon Tyne NE4 5PL, United Kingdom
19. Population Health Sciences Institute, Department of Medicine, Newcastle University, Newcastle upon Tyne, United Kingdom

**E-mail address of corresponding author:**
edcarrol@liverpool.ac.uk

## Appendix A – Hospital characteristics

| Hospital | City, country | Type of hospital | Annual pediatric ED visits | Primary care during OOH | Self-referrals | GP referrals |
|---|---|---|---|---|---|---|
| Radboud University Medical Center (N=677) | Nijmegen, the Netherlands (NL1) | University | <10,000 | Yes | 4% | 40% |
| Erasmus MC – Sophia Children's Hospital (N=1683) | Rotterdam, the Netherlands (NL2) | University | <10,000 | Yes | 22% | 20% |
| Univerzitetni klinicni center (N=3667) | Ljubljana, Slovenia | University | <10,000 | Yes | 1% | 13% |
| Canisius Wilhelmina Ziekenhuis (N=423) | Nijmegen, the Netherlands (NL3) | Teaching | <10,000 | Yes | 1% | 90% |
| Medizinische Universität Graz (N=2241) | Graz, Austria | University | 10,000-30,000 | No | 43% | 4% |
| P. and A. Kyriakou Children's Hospital (N=4548) | Athens, Greece | University | >30,000 | No | 92% | 0.1% |
| St. Mary's Hospital (N=5714) | London, UK (UK3) | University | >30,000 | Yes | 54% | 4% |
| Hospital Clínico Universitario (N=3877) | Santiago de Compostela, Spain | University | >30,000 | Yes | 86% | 12% |
| Children's Clinical University Hospital (N=9000) | Riga, Latvia | University | >30,000 | No | 53% | 4% |
| Alder Hey Children's Hospital (N=1623) | Liverpool, UK (UK1) | University | >30,000 | Yes | 66% | 16% |
| Dr. von Hauner Children's Hospital (N=1173) | Munich, Germany | University | 10,000-30,000 | Yes | 95% | 0.3% |
| Great North Children's Hospital (N=3854) | Newcastle, UK (UK2) | University | >30,000 | Yes | 60% | 11% |

## Appendix B - Phenotyping algorithm PERFORM

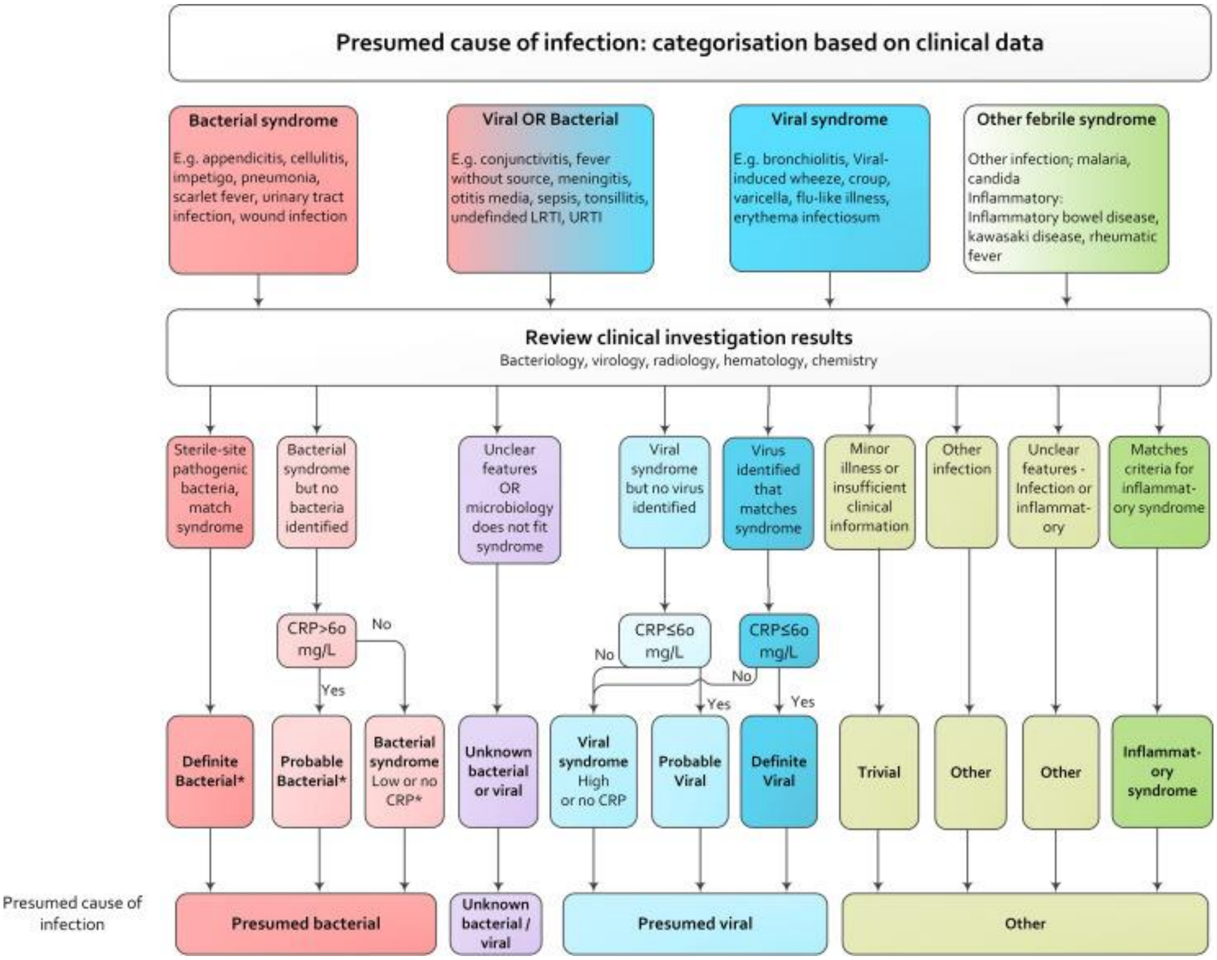


*In case of viral co-infection, bacterial infection got priority

**Appendix C – Immediate life-saving interventions**

| **ILSI** | **Procedures** |
|---|---|
| 1. Airway and breathing support | Intubation, non-invasive ventilation, non-rebreathing mask, nasal cannula for oxygen |
| 2. Electrical therapy | Defibrillation, emergent cardioversion, external pacing |
| 3. Emergency procedures | Chest needle decompression, pericardiocentesis, open thoracotomy |
| 4. Hemodynamic support | Significant intravenous or intraosseous fluid resuscitation, blood administration, control of major bleeding |
| 5. Emergency medication | Atropine, adenosine, epinephrine, naloxone or inotropes |

## Appendix D - Questionnaire and corresponding answers on EMS per country during the MOFICHE study period 2017-2018

Could you please answer the questions below? Please select all answer options that apply.

**Q1) How does the process look like, starting from the call to the emergency number until arrival at the hospital by EMS?**

**Q2) Who can call the emergency number for EMS transport?**

a) Doctors
b) Parents
c) Other, please specify

**Q3) What happens when the EMS arrive at the patient?**

a) The patient is always transported by EMS
b) They assess/triage the patient first and then decide whether they transport the patient by EMS
c) Other, please specify

**Q4) Where can EMS transport the patients to?**

a) The patient is always transported to the ED
b) The patient can be transported to a GP out of office
c) The EMS team can decide to let the patient stay at home
d) Other, please specify

**Q5) What are in practice the reasons for EMS use?**

a) Transport of critically ill patients
b) Lack of private transport
c) Emergency call during out of office hours
c) Other, please specify

**Answers:**

| | **Q1** |
|---|---|
| **The Netherlands** | Everyone can directly call the emergency number and the caller is asked to explain the medical situation of the patient. The staff at the operation center can advise to call the GP (out of office) when appropriate. When EMS arrive at the patient they triage the patient first and then decide whether the patient need to be transported to the hospital or they can advise the patient to go to the GP (out of office). When arriving at the ED the patients are directly transported to the examination room, where the EMS staff present the patient to the physicians at the ED and the patients' vital signs and medical procedures they already performed. |

| | |
|---|---|
| **Spain** | Anyone can call directly to the emergency number. A specific trained subject will attend the call and determine whether an effective should go directly to the address or if the issue may be sorted by phone. If the ambulance goes to the indicated address and the subject is attended and problem solved, the process ends here; otherwise, the subject will be transferred to the hospital in a medical ambulance and treated if necessary during the transport. |
| **Austria** | It is run by various private companies, mainly the red cross, but also other like green cross. Those companies provide emergency transports but also regular patient transports. Emergency transports include at least one emergency paramedic. Depending on the severity, an additional emergency doctor can be included, either upfront or at arrival of the paramedic. The emergency doctor always comes with a separate car and driver. |
| **Greece** | After the call to the emergency number, the staff at the operation centre asks for some information about the patient to evaluate the severity and give priority to critically ill patients. But eventually any patient will be transferred to ED, even with less severe symptoms, as long as the parents are patient enough to wait. |
| **UK** | In case of a serious medical emergency anyone can call 999. When the EMS are going to the patient a call number will be made. EMS staff will assess the patient first and decide whether transport to the hospital is needed. If they decide on transporting the patient to the hospital they will report this via the call center. |
| **Slovenia** | The call to 112 is triaged and the need for EMS is established by qualified healthcare personnel at the dispatch centre. We have different types of mobile units – a reanimobile (with a doctor, nurse and paramedic for medical interventions at the site of the incident), emergency vehicle (nurse and paramedic - for transport of patients), motorbike (nurse), helicopter (doctor and nurse - for transport of critically ill patients). The type of help (could be a combination) that will be deployed is decided at the dispatch center. When the EMS arrives, they asses the patient, apply treatment if necessary and transport to the nearest hospital with an appropriate ED which they notify about their upcoming arrival. |
| **Latvia** | Operator asks the first red flag questions - if emergency they will send an ambulance car in a define time depending on emergency situation. If not so acute, may transfer to consultation line. |
| **Germany** | When calling the emergency number the caller is asked to shortly explain the (current) medical situation of the person in question. Depending on the situation the emergency dispatcher may stay on the phone in order to assist the caller. When EMS arrive they triage the patient and decide whether there are medical procedures that need to be performed on the spot before transportation, whether the patient needs to be transported to the ED or whether they may also remain where they are. On the way to the ED patients vitals´ are being monitored by EMS. If possible EMS also ask about history of current and prior medical illness and medication. Upon arrival at the ED the patients are almost always directly transported to an examination room, where EMS present the doctor with the priory collected information. |

| | Q2 | Q3 | Q4 | Q5 |
|---|---|---|---|---|
| **The Netherlands** | Anyone | EMS staff triage the patient first and decide whether transport by EMS is needed. | The patient is always transported to the ED. The EMS team can decide to let the patient stay at home or advice | Transport of critically ill patients. Lack of private transport. Emergency call during out of office hours. |

| | | | | |
|---|---|---|---|---|
| | | | the patient to go the GP (out of office). | |
| **Spain** | Doctors, parents, any person eg the school director. | They assess/triage the patient first and then decide whether they transport the patient by EMS. | Patient not always transported to the ED, only if needed. Patient can be transported to GP out of office.<br>The EMS team can decide to let the patient stay at home. | Transport of critically ill patients. Lack of private transport. Emergency call during out of office hours. |
| **Austria** | Doctors, mainly parents, anyone | The patient is almost always transported by EMS, except for patients that refuse transport (e.g. some alcohol intoxication with only mild intoxication). | Patient is always transported to the ED. The Ems can decide to let the patient stay at home if the Ems is accompanied by an emergency doctor. | Transport of critically ill patients. Lack of private transport in case of emergency, emergency call during out of office hours. |
| **Greece** | Anyone can call (doctors, parents) | The patient is always transported by EMS. | The patient is always transported to the ED. | Transport of critically ill patients. Lack of private transport. |
| **UK** | Everyone can call 999 | They assess/triage the patient first and then decide whether they transport the patient by EMS. EMS cannot refuse to transport patients under the age of 1. | The patient is always transported to the ED.<br>The EMS team can decide to let the patient stay at home. | Transport of critically ill patients, Lack of private transport, Emergency call during out of office hours, hospital to hospital transport. |
| **Slovenia** | Doctors, parents, anyone that thinks there is need for EMS (ie 112) | They assess/triage the patient first and then decide whether they transport the patient by EMS – but it's very rare that they don't transport them, as the triage has already been done at the dispatch center. | The patient is always transported to the ED.<br>The EMS team can decide to let the patient stay at home (but this occurs only rarely). | Transport of critically ill patients.<br>Lack of private transport - the dispatch center also allocates resources (vehicles & personnel) for non-emergency transport of patients<br>Emergency call during out of office hours. |
| **Latvia** | Doctors, parents, everyone | Mostly always children below 1 year of age are transported to the | The patient is always transported to the ED. The EMS team can decide to | Transport of critically ill patients. But in reality also in case |

| | | | | |
|---|---|---|---|---|
| | | ED otherwise not necessarily at all. They assess/triage the patient first and then decide whether they transport the patient by EMS. | let the patient stay at home. | of lack of private transport and emergency call during out of office hours. |
| **Germany** | Doctors, parents, everyone | They assess/triage the patient first and then decide whether they transport the patient by EMS, if the patient/guardian insist even if the EMS don't triage the patient as someone in need of EMS transportation. | The patient is always transported to the ED, the EMS team can decide to let the patient stay at home, from one hospital to another hospital. | Transport of critically ill patients, lack of private transport, emergency call during out of office hours. |

**Appendix E – Diagram of healthcare pathways initiating EMS transport to ED**

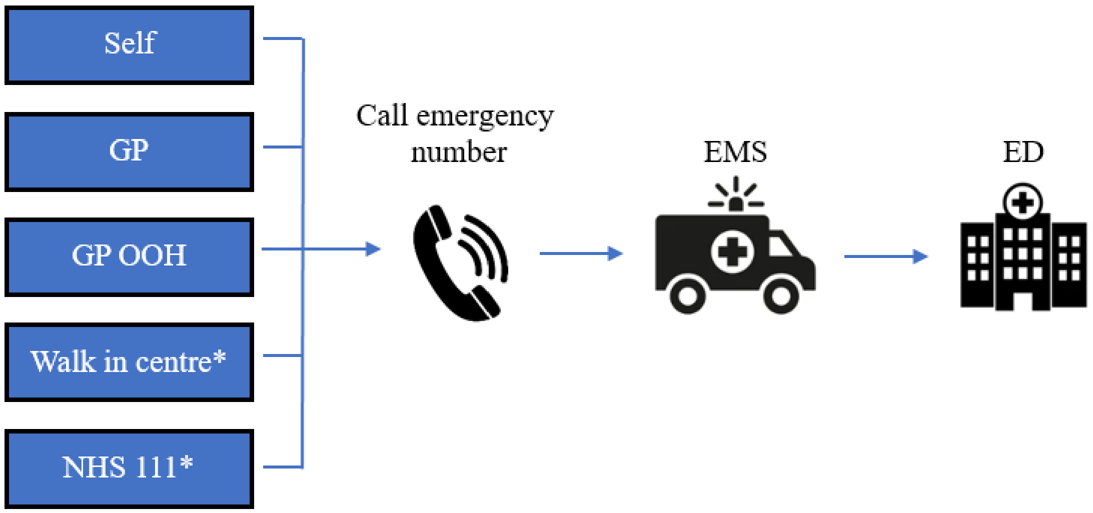


*Health care providers in UK
GP OOH = GP out of office hours, not present in all settings

**Appendix F – Range of EMS use and discordant EMS use across the EDs in absolute numbers and percentages (%)**

| **Hospital** | **Transport by EMS*** | **Discordant EMS use**∞ |
|---|---|---|
| *Total (N=36,156)* | *5464 (15)* | *1601 (29)* |
| NL1 (N=664) | 70 (11) | 1 (1) |
| NL2 (N=1422) | 171 (12) | 4 (2) |
| Slovenia (N=3656) | 288 (8) | 23 (8) |
| NL3 (N=404) | 16 (4) | 2 (13) |
| Austria (N=1241) | 63 (5) | 9 (14) |
| Greece (N=4513) | 6 (0.1) | 1 (17) |
| UK3 (N=5678) | 380 (7) | 66 (17) |
| Spain (N=3864) | 69 (2) | 16 (23) |
| Latvia (N=8994) | 3740 (42) | 1106 (30) |
| UK1 (N=1589) | 95 (6) | 38 (40) |
| Germany (N=830) | 9 (1) | 4 (44) |
| UK2 (N=3301) | 557 (17) | 331 (59) |

Table is ordered according increasing percentages of discordant EMS use

* Denominator total visits per ED setting

∞ Denominator transport by EMS per ED setting

**Appendix G – Odds ratios and adjusted odds ratios of logistic regression analysis**

Association between markers of urgency and EMS use

| | **OR (95% CI)** | **aOR (95% CI)** |
|---|---|---|
| **High/intermediate triage urgency** | 2.7 (2.5-2.8) | 3.5 (3.3-3.7) |
| **Advanced diagnostics** | 1.2 (1.1-1.3) | 1.2 (1.0-1.3) |
| **Immediate life-saving interventions** | 3.0 (2.6-3.6) | 4.5 (3.8-5.4) |
| **O2 therapy** | 1.6 (1.4-1.9) | 1.7 (1.5-2.0) |
| **Admission** | 1.8 (1.7-1.9) | 1.7 (1.6-1.8) |

Non-EMS group as reference group.

Adjusted for age, gender visiting hours, presenting symptoms, ED setting.

**Appendix H – Sensitivity analysis for discordant EMS use**

| **Hospital** | **Transport by EMS 100%** | **Transport by EMS 80%** | **Discordant EMS use in 80%*** | **Discordant EMS use in 100%∞** |
|---|---|---|---|---|
| *Total (N=36,156)* | *5464* | *4335* | *1261 (29%)* | *1601 (29%)* |
| NL1 (N=664) | 70 | 52 | 1 (2) | 1 (1) |
| NL2 (N=1422) | 171 | 140 | 4 (3) | 4 (2) |
| Slovenia (N=3656) | 288 | 227 | 18 (8) | 23 (8) |
| NL3 (N=404) | 16 | 14 | 1 (7) | 2 (13) |
| Austria (N=1241) | 63 | 43 | 6 (14) | 9 (14) |
| Greece (N=4513) | 6 | 5 | 1 (20) | 1 (17) |
| UK3 (N=5678) | 380 | 307 | 50 (16) | 66 (17) |
| Spain (N=3864) | 69 | 58 | 12 (21) | 16 (23) |
| Latvia (N=8994) | 3740 | 2956 | 871 (30) | 1106 (30) |
| UK1 (N=1589) | 95 | 74 | 33 (45) | 38 (40) |
| Germany (N=830) | 9 | 7 | 4 (43) | 4 (44) |
| UK2 (N=3301) | 557 | 448 | 262 (59) | 331 (59) |

* Denominator transport by EMS per ED setting in the column with 80%

∞ Denominator transport by EMS per ED setting in the column with 100%

**Appendix I – Age distribution per ED setting in EMS group and in discordant EMS group**

| EMS use (N=5464) | Median age in years (IQR 25-75) |
|---|---|
| NL1 (N=70) | 2.5 (1.7-5.4) |
| NL2 (N=171) | 2.8 (1.6-5.5) |
| Slovenia (N=288) | 1.9 (1.2-3.1) |
| NL3 (N=16) | 1.5 (1.2-2.9) |
| Austria (N=63) | 1.8 (1.3-4.6) |
| Greece (N=6) | 4.9 (3.1-7.0) |
| UK3 (N=380) | 2.3 (1.2-4.4) |
| Spain (N=69) | 2.0 (1.2-4.0) |
| Latvia (N=3740) | 2.6 (1.3-5.3) |
| UK1 (N=95) | 2.4 (1.1-5.0) |
| Germany (N=9) | 5.7 (2.9-7.8) |
| UK2 (N=557) | 2.8 (1.6-4.0) |

Range median age: 1,5-5,7 years

| Discordant EMS use (N=1601) | Median age in years (IQR 25-75) |
|---|---|
| NL1 (N=1) | 14.1 (14.1-14.1) |
| NL2 (N=4) | 3.2 (2.3-3.5) |
| Slovenia (N=23) | 2.4 (1.5-4.7) |
| NL3 (N=2) | 1.4 (1.3-1.4) |
| Austria (N=9) | 3.2 (1.2-5.6) |
| Greece (N=1) | 4.6 (4.6-4.6) |
| UK3 (N=66) | 3.0 (1.3-4.4) |
| Spain (N=16) | 2.4 (1.2-8.3) |
| Latvia (N=1106) | 2.6 (1.3-5.3) |
| UK1 (N=38) | 2.3 (1.1-4.4) |
| Germany (N=4) | 6.1 (3.2-7.7) |
| UK2 (N=331) | 2.0 (1.1-3.8) |

Range median age: 1,4 – 14,1 years

## Appendix J – PERFORM consortium authors list

PARTNER: IMPERIAL COLLEGE (UK)

Chief investigator/PERFORM coordinator:

Michael Levin

Principal and co-investigators; work package leads (alphabetical order)

Aubrey Cunnington (grant application)

Tisham De (work package lead)

Jethro Herberg (Principle Investigator, Deputy Coordinator, grant application)

Myrsini Kaforou (grant application, work package lead)

Victoria Wright (grant application, Scientific Coordinator)

Research Group (alphabetical order)

Lucas Baumard; Evangelos Bellos; Giselle D'Souza; Rachel Galassini; Dominic Habgood-Coote; Shea Hamilton; Clive Hoggart; Sara Hourmat; Heather Jackson; Ian Maconochie; Stephanie Menikou; Naomi Lin; Samuel Nichols; Ruud Nijman; Ivonne Pena Paz; Priyen Shah; Ching-Fen Shen; Ortensia Vito; Clare Wilson

Clinical recruitment at Imperial College Healthcare NHS Trust (alphabetical order))

Amina Abdulla; Ladan Ali; Sarah Darnell; Rikke Jorgensen; Sobia Mustafa; Salina Persand

Imperial College Faculty of Engineering

Molly Stevens (co-investigator), Eunjung Kim (research group); Benjamin Pierce (research group)

Clinical recruitment at Brighton and Sussex University Hospitals

Katy Fidler (Principle Investigator)

Julia Dudley (Clinical Research Registrar)

Research nurses: Vivien Richmond, Emma Tavliavini

Clinical recruitment at National Cheng Kung University Hospital

Ching-Fen Shen (Principal Investigator); Ching-Chuan Liu (Co-investigator); Shih-Min Wang (Co-investigator), funded by the Center of Clinical Medicine Research, National Cheng Kung University

**SERGAS Partner (Spain)**
Principal Investigators
Federico Martinón-Torres[1]
Antonio Salas[1,2]

GENVIP RESEARCH GROUP (in alphabetical order):
Fernando Álvez González[1], Cristina Balo Farto[1], Ruth Barral-Arca[1,2], María Barreiro Castro[1], Xabier Bello[1,2], Mirian Ben García[1], Sandra Carnota[1], Miriam Cebey-López[1], María José Curras-Tuala[1,2], Carlos Durán Suárez[1], Luisa García Vicente[1], Alberto Gómez-Carballa[1,2], Jose Gómez Rial[1], Pilar Leboráns Iglesias[1], Federico Martinón-Torres[1], Nazareth Martinón-Torres[1], José María Martinón Sánchez[1], Belén Mosquera Pérez[1], Jacobo Pardo-Seco[1,2], Lidia Piñeiro Rodríguez[1], Sara Pischedda[1,2], Sara Rey Vázquez[1], Irene Rivero Calle[1], Carmen Rodríguez-Tenreiro[1], Lorenzo Redondo-Collazo[1], Miguel Sadiki Ora[1], Antonio Salas[1,2], Sonia Serén Fernández[1], Cristina Serén Trasorras[1], Marisol Vilas Iglesias[1].

[1] Translational Pediatrics and Infectious Diseases, Pediatrics Department, Hospital Clínico Universitario de Santiago, Santiago de Compostela, Spain, and GENVIP Research Group (www.genvip.org), Instituto de Investigación Sanitaria de Santiago, Universidad de Santiago de Compostela, Galicia, Spain.
[2] Unidade de Xenética, Departamento de Anatomía Patolóxica e Ciencias Forenses, Instituto de Ciencias Forenses, Facultade de Medicina, Universidade de Santiago de Compostela, and GenPop Research Group, Instituto de Investigaciones Sanitarias (IDIS), Hospital Clínico Universitario de Santiago, Galicia, Spain
[3] Fundación Pública Galega de Medicina Xenómica, Servizo Galego de Saúde (SERGAS), Instituto de Investigaciones Sanitarias (IDIS), and Grupo de Medicina Xenómica, Centro de Investigación Biomédica en Red de Enfermedades Raras (CIBERER), Universidade de Santiago de Compostela (USC), Santiago de Compostela, Spain

**RSU Partner (Latvia)**
Principal Investigator
Dace Zavadska[1,2]

Other RSU group authors (in alphabetical order):

Anda Balode[1,2], Arta Bārzdiņa[1,2], Dārta Deksne[1,2], Dace Gardovska[1,2], Dagne Grāvele[2], Ilze Grope[1,2], Anija Meiere[1,2], Ieva Nokalna[1,2], Jana Pavāre[1,2], Zanda Pučuka[1,2], Katrīna Selecka[1,2], Aleksandra Sidorova[1,2], Dace Svile[2], Urzula Nora Urbāne[1,2].

[1] Riga Stradins university, Riga, Latvia.
[2] Children clinical university hospital, Riga, Latvia.

**Medical Research Council Unit The Gambia (MRCG) at LSHTM Partner**

**Principal Investigator**
Effua Usuf

**Additional Investigators**
Kalifa Bojang
Syed M. A. Zaman
Fatou Secka
Suzanne Anderson
Anna RocaIsatou Sarr
Momodou Saidykhan
Saffiatou Darboe
Samba Ceesay
Umberto D'alessandro

Medical Research Council Unit The Gambia at LSHTM
P O Box 273,
Fajara, The Gambia

**ERASMUS MC-Sophia Children's Hospital**

Principal Investigator
Henriëtte A. Moll[1]

Research group
Dorine M. Borensztajn[1], Nienke N. Hagedoorn, Chantal Tan [1], [1], Clementien L. Vermont[2], Joany Zachariasse [1]

Additional investigator
W Dik [3]

[1] Erasmus MC-Sophia Children's Hospital, Department of General Paediatrics, Rotterdam, the Netherlands

[2] Erasmus MC-Sophia Children's Hospital, Department of Paediatric Infectious Diseases & Immunology, Rotterdam, the Netherlands
[3] Erasmus MC, Department of immunology, Rotterdam, the Netherlands

**Swiss Pediatric Sepsis Study**

**Principal Investigators:**

Philipp Agyeman, MD [1] (ORCID 0000-0002-8339-5444), Luregn J Schlapbach, MD, FCICM [2,3] (ORCID 0000-0003-2281-2598)

**Clinical recruitment at University Children's Hospital Bern for PERFORM:**

Christoph Aebi [1], Verena Wyss [1], Mariama Usman [1]

**Principal and co-investigators for the Swiss Pediatric Sepsis Study:**

Philipp Agyeman, MD [1], Luregn J Schlapbach, MD, FCICM [2,3], Eric Giannoni, MD [4,5], Martin Stocker, MD [6], Klara M Posfay-Barbe, MD [7], Ulrich Heininger, MD [8], Sara Bernhard-Stirnemann, MD [9], Anita Niederer-Loher, MD [10], Christian Kahlert, MD [10], Giancarlo Natalucci, MD [11], Christa Relly, MD [12], Thomas Riedel, MD [13], Christoph Aebi, MD [1], Christoph Berger, MD [12] **for the Swiss Pediatric Sepsis Study**

**Affiliations:**

[1] Department of Pediatrics, Inselspital, Bern University Hospital, University of Bern, Switzerland

[2] Neonatal and Pediatric Intensive Care Unit, Children's Research Center, University Children's Hospital Zurich, University of Zurich, Zurich, Switzerland

[3]Child Health Research Centre, University of Queensland, and Queensland Children`s Hospital, Brisbane, Australia

[4] Clinic of Neonatology, Department Mother-Woman-Child, Lausanne University Hospital and University of Lausanne, Switzerland

[5] Infectious Diseases Service, Department of Medicine, Lausanne University Hospital and University of Lausanne, Switzerland

[6] Department of Pediatrics, Children's Hospital Lucerne, Lucerne, Switzerland

[7] Pediatric Infectious Diseases Unit, Children's Hospital of Geneva, University Hospitals of Geneva, Geneva, Switzerland

[8] Infectious Diseases and Vaccinology, University of Basel Children's Hospital, Basel, Switzerland

[9] Children's Hospital Aarau, Aarau, Switzerland

[10] Division of Infectious Diseases and Hospital Epidemiology, Children's Hospital of Eastern Switzerland St. Gallen, St. Gallen, Switzerland

[11] Department of Neonatology, University Hospital Zurich, Zurich, Switzerland

[12] Division of Infectious Diseases and Hospital Epidemiology, and Children's Research Center, University Children's Hospital Zurich, Switzerland

[13] Children's Hospital Chur, Chur, Switzerland

**Liverpool Partner**

Principal Investigators

Enitan D Carrol[1,2,3]

Stéphane Paulus [1,]

Research Group (in alphabetical order):

Elizabeth Cocklin[1], Rebecca Jennings[4], Joanne Johnston[4], Simon Leigh[1], Karen Newall[4], Sam Romaine[1]

[1] Department of Clinical Infection, Microbiology and Immunology, University of Liverpool Institute of Infection and Global Health , Liverpool, England
[2] Alder Hey Children's Hospital, Department of Infectious Diseases, Eaton Road, Liverpool, L12 2AP
[3] Liverpool Health Partners, 1st Floor, Liverpool Science Park, 131 Mount Pleasant, Liverpool, L3 5TF

[4]Alder Hey Children's Hospital, Clinical Research Business Unit, Eaton Road, Liverpool, L12 2AP

**NKUA Partner (Greece)**

Principal investigator: Professor **Maria Tsolia** (all activities)

Investigator/Research fellow: **Irini Eleftheriou** (all activities)

Additional investigators:

Recruitment: Maria Tambouratzi

Lab: Antonis Marmarinos (Quality Manager)

Lab: Marietta Xagorari

Kelly Syggelou

2nd Department of Pediatrics, National and Kapodistrian University of Athens,

“P. and A. Kyriakou” Children’s Hospital

Thivon and Levadias

Goudi, Athens

**Micropathology Ltd :**

Principal Investigator:

Professor Colin Fink[1], Clinical Microbiologist

Additional investigators

Dr Marie Voice[1], Post doc scientist

Dr. Leo Calvo-Bado[1], Post doc scientist

[1] Micropathology Ltd, The Venture Center, University of Warwick Science Park, Sir William Lyons Road, Coventry, CV4 7EZ.

**Medical University of Graz, Austria (MUG)**

Principal Investigator:

Werner Zenz[1] (all activities)

Co-investigators (in alphabetical order)

Benno Kohlmaier[1] (all activities)

Nina A. Schweintzger[1] (all activities)

Manfred G. Sagmeister[1] (study design, consortium wide sample management)

Research team

Daniela S. Kohlfürst[1] (study design)

Christoph Zurl[1] (BIVA PIC)

Alexander Binder[1] (grant application)

Recruitment team, data managers, (in alphabetical order):

Susanne Hösele[1], Manuel Leitner[1], Lena Pölz[1], Glorija Rajic[1],

Clinical recruitment partners (in alphabetical order):

Sebastian Bauchinger[1], Hinrich Baumgart[4], Martin Benesch[3], Astrid Ceolotto[1], Ernst Eber[2], Siegfried Gallistl[1], Gunther Gores[5], Harald Haidl[1], Almuthe Hauer[1], Christa Hude[1], Markus Keldorfer[5], Larissa Krenn[4], Heidemarie Pilch[5], Andreas Pfleger[2], Klaus Pfurtscheller[4], Gudrun Nordberg[5], Tobias Niedrist[8], Siegfried Rödl[4], Andrea Skrabl-Baumgartner[1], Matthias Sperl[7], Laura Stampfer[5], Volker Strenger[3], Holger Till[6], Andreas Trobisch[5], Sabine Löffler[5]

Author Affiliations:

[1] Department of Pediatrics and Adolescent Medicine, Division of General Pediatrics, Medical University of Graz, Graz, Austria

[2]Department of Pediatric Pulmonology, Medical University of Graz, Graz, Austria

[3]Department of Pediatric Hematooncoloy, Medical University of Graz, Graz, Austria

[4]Paediatric Intensive Care Unit, Medical University of Graz, Graz, Austria

[5]University Clinic of Paediatrics and Adolescent Medicine Graz, Medical University Graz, Graz,Austria

[6]Department of Paediatric and Adolescence Surgery, Medical University Graz, Graz, Austria

[7]Department of Pediatric Orthopedics, Medical University Graz, Graz, Austria

[8]Clinical Institute of Medical and Chemical Laboratory Diagnostics, Medical University Graz, Graz, Austria

**London School of Hygiene and Tropical Medicine**

**WP 1 WP2, WP5**

Principal Investigator:

Dr Shunmay Yeung[1,2 3] PhD, MBBS, FRCPCH, MRCP, DTM&H

Research Group

Dr Juan Emmanuel Dewez[1] MD, DTM&H, MSc

Prof Martin Hibberd [1] BSc, PhD

Mr David Bath[2] MSc, MAppFin, BA(Hons)

Dr Alec Miners[2] BA(Hons), MSc, PhD

Dr Ruud Nijman[3] PhD MSc MD MRCPCH

Dr Catherine Wedderburn[1] BA, MBChB, DTM&H, MSc, MRCPCH

Ms Anne Meierford[1] MSc, BMedSc, BMBS

Dr Baptiste Leurent[4], PhD, MSc

1. Faculty of Infectious and Tropical Disease, London School of Hygiene and Tropical Medicine, London, UK
2. Faculty of Public Health and Policy, London School of Hygiene and Tropical Medicine, London, UK
3. Department of Paediatrics, St. Mary's Hospital Imperial College Hospital, London, UK
4. Faculty of Epidemiology and Population Health, London School of Hygiene and Tropical Medicine, London, UK

**Radboud University Medical Center (RUMC), The Netherlands**

Principal Investigators:

Ronald de Groot [1], Michiel van der Flier [1,2,3], Marien I. de Jonge[1]

Co-investigators Radboud University Medical Center (in alphabetical order):

Koen van Aerde[1,2], Wynand Alkema[1], Bryan van den Broek[1], Jolein Gloerich[1], Alain J. van Gool[1], Stefanie Henriet[1,2], Martijn Huijnen[1], Ria Philipsen[1], Esther Willems[1]

Investigators PeDBIG PERFORM DUTCH CLINICAL NETWORK (in alphabetical order):

G.P.J.M. Gerrits[8], M. van Leur[8,] J. Heidema [4],L. de Haan[1,2] C.J. Miedema [5], C. Neeleman [1] C.C. Obihara [6], G.A. Tramper-Stranders7[6]

1. Radboud University Medical Center, Nijmegen, The Netherlands
2. Amalia Children's Hospital, Nijmegen, The Netherlands
3. Wilhelmina Children's Hospital, University Medical Center Utrecht, Utrecht, The Netherlands
4. St. Antonius Hospital, Nieuwegein, The Netherlands
5. Catharina Hospital, Eindhoven, The Netherlands
6. ETZ Elisabeth, Tilburg, The Netherlands
7. Franciscus Gasthuis, Rotterdam, The Netherlands
8. Canisius Wilhelmina Hospital, Nijmegen, The Netherlands

**Oxford team (UK)**

Principal Investigators

Andrew J. Pollard[1,2], Rama Kandasamy[1,2], Stéphane Paulus [1,2]

Additional Investigators

Michael J. Carter[1,2], Daniel O'Connor[1,2], Sagida Bibi[1,2], Dominic F. Kelly[1,2], Meeru Gurung[3], Stephen Thorson[3], Imran Ansari[3], David R. Murdoch[4], Shrijana Shrestha[3], Zoe Oliver[5]

Author Affiliations:

[1]Oxford Vaccine Group, Department of Paediatrics, University of Oxford, Oxford, United Kingdom.

[2]NIHR Oxford Biomedical Research Centre, Oxford, United Kingdom.

[3]Paediatric Research Unit, Patan Academy of Health Sciences, Kathmandu, Nepal.

[4]Department of Pathology, University of Otago, Christchurch, New Zealand.

[5] Department of Paediatrics, University of Oxford.

**Newcastle University, Newcastle upon Tyne, (UK)**

Principal Investigator:

Marieke Emonts [1,2,3] (all activities)

Co-investigators

Emma Lim[2,3,7] (all activities)

Lucille Valentine[4]

Recruitment team (alphabetical), data-managers, and GNCH Research unit:

Karen Allen[5], Kathryn Bell[5], Adora Chan[5], Stephen Crulley[5], Kirsty Devine[5], Daniel Fabian[5], Sharon King[5], Paul McAlinden[5], Sam McDonald[5], Anne McDonnell2,[5], Ailsa Pickering[2,5], Evelyn Thomson[5], Amanda Wood[5], Diane Wallia[5], Phil Woodsford[5],

Sample processing: Frances Baxter[5], Ashley Bell[5], Mathew Rhodes[5]

PICU recruitment

Rachel Agbeko[8]

Christine Mackerness[8]

Students MOFICHE

Bryan Baas[2], Lieke Kloosterhuis[2], Wilma Oosthoek[2]

Students/medical staff PERFORM

Tasnim Arif[6], Joshua Bennet[2], Kalvin Collings[2], Ilona van der Giessen[2], Alex Martin[2], Aqeela Rashid[6], Emily Rowlands[2], Gabriella de Vries[2], Fabian van der Velden[2]

Engagement work/ethics/cost effectiveness

Lucille Valentine [4], Mike Martin[9], Ravi Mistry[2], Lucille Valentine[4]

Author Affiliations:

[1] Translational and Clinical Research Institute, Newcastle University, Newcastle upon Tyne UK

[2]Great North Children's Hospital, Paediatric Immunology, Infectious Diseases & Allergy, Newcastle upon Tyne Hospitals NHS Foundation Trust, Newcastle upon Tyne, United Kingdom.

[3]NIHR Newcastle Biomedical Research Centre based at Newcastle upon Tyne Hospitals NHS Trust and Newcastle University, Westgate Rd, Newcastle upon Tyne NE4 5PL, United Kingdom

[4]Newcastle University Business School, Centre for Knowledge, Innovation, Technology and Enterprise (KITE), Newcastle upon Tyne, United Kingdom

[5]Great North Children's Hospital, Research Unit, Newcastle upon Tyne Hospitals NHS Foundation Trust, Newcastle upon Tyne, United Kingdom.

[6]Great North Children's Hospital, Paediatric Oncology, Newcastle upon Tyne Hospitals NHS Foundation Trust, Newcastle upon Tyne, United Kingdom.

[7]Population Health Sciences Institute, Newcastle University, Newcastle upon Tyne, UK

[8]Great North Children's Hospital, Paediatric Intensive Care Unit, Newcastle upon Tyne Hospitals NHS Foundation Trust, Newcastle upon Tyne, United Kingdom.

[9]Northumbria University, Newcastle upon Tyne, United Kingdom.

**LMU Munich Partner (Germany)**

Principal Investigator:

Ulrich von Both[1,2] MD, FRCPCH (all activities)

Research group:

Laura Kolberg[1] MSc (all activities)

Manuela Zwerenz[1] MSc, Judith Buschbeck[1] PhD

Clinical recruitment partners (in alphabetical order):

Christoph Bidlingmaier[3], Vera Binder[4], Katharina Danhauser[5], Nikolaus Haas[10], Matthias Griese[6], Tobias Feuchtinger[4], Julia Keil[9], Matthias Kappler[6], Eberhard Lurz[7], Georg Muench[8], Karl Reiter[9], Carola Schoen[9]

Author Affiliations:

[1]Div. Paediatric Infectious Diseases, Hauner Children's Hospital, University Hospital, Ludwig Maximilians University (LMU), Munich, Germany

[2]German Center for Infection Research (DZIF), Partner Site Munich, Munich, Germany

[3]Div. of General Paediatrics, [4]Div. Paediatric Haematology & Oncology, [5]Div. of Paediatric Rheumatology, [6]Div. of Paediatric Pulmonology, [7]Div. of Paediatric Gastroenterology, [8]Neonatal Intensive Care Unit, [9]Paediatric Intensive Care Unit Hauner Children's Hospital, University Hospital, Ludwig Maximilians University (LMU), Munich, Germany, [10]Department Pediatric Cardiology and Pediatric Intensive Care, University Hospital, Ludwig Maximilians University (LMU), Munich, Germany

**bioMérieux, France**

Principal Investigator:
François Mallet[1,2, 3]
Research Group:
Karen Brengel-Pesce[1,2, 3]
Alexandre Pachot[1]
Marine Mommert[1,2]

*[1]Open Innovation & Partnerships (OIP), bioMérieux S.A., Marcy l'Etoile, France*
*[2] Joint research unit Hospice Civils de Lyon - bioMérieux, Centre Hospitalier Lyon Sud, 165 Chemin du Grand Revoyet, 69310 Pierre-Bénite, France*
*[3] EA 7426 Pathophysiology of Injury-induced Immunosuppression, University of Lyon1-Hospices Civils de Lyon-bioMérieux, Hôpital Edouard Herriot, 5 Place d'Arsonval, 69437 Lyon Cedex 3, France*

**Department of Infectious Diseases, University Medical Centre Ljubljana, Slovenia**

Principal Investigator:

Marko Pokorn[1,2,3] MD, PhD

Research Group:

Mojca Kolnik[1] MD, Katarina Vincek[1] MD, Tina Plankar Srovin[1] MD, PhD, Natalija Bahovec[1] MD, Petra Prunk[1] MD, Veronika Osterman[1] MD, Tanja Avramoska[1] MD

Affiliations:

[1]Department of Infectious Diseases, University Medical Centre Ljubljana, Japljeva 2, SI-1525 Ljubljana, Slovenia

[2]University Childrens' Hospital, University Medical Centre Ljubljana, Ljubljana, Slovenia

[3]Department of Infectious Diseases and Epidemiology, Faculty of Medicine, University of Ljubljana, Slovenia

**Amsterdam, Academic Medical Hospital & Sanquin Research Institute (NL)**

Principal Investigator:

Taco Kuijpers [1,2]

Co-investigators

Ilse Jongerius [2]

Recruitment team (EUCLIDS, PERFORM):

J.M. van den Berg[1], D. Schonenberg[1], A.M. Barendregt[1], D. Pajkrt[1], M. van der Kuip[1,3], A.M. van Furth[1,3]

Students PERFORM

Evelien Sprenkeler [2], Judith Zandstra [2],

Technical support PERFORM

G. van Mierlo [2], J. Geissler [2]

Author Affiliations:

[1] Amsterdam University Medical Center (Amsterdam UMC), location Academic Medical Center (AMC), Dept of Pediatric Immunology, Rheumatology and Infectious Diseases, University of Amsterdam, Amsterdam, the Netherlands

[2] Sanquin Research Institute, & Landsteiner Laboratory at the AMC, University of Amsterdam, Amsterdam, the Netherlands.

[3] Amsterdam University Medical Center (Amsterdam UMC), location Vrije Universiteit Medical Center (VUMC), Dept of Pediatric Infectious Diseases and Immunology, Free University (VU), Amsterdam, the Netherlands (former affiliation)
